# Therapeutic Cardiac‐Targeted Delivery of *miR‐1* Reverses Pressure Overload–Induced Cardiac Hypertrophy and Attenuates Pathological Remodeling

**DOI:** 10.1161/JAHA.113.000078

**Published:** 2013-04-24

**Authors:** Ioannis Karakikes, Antoine H. Chaanine, Soojeong Kang, Bertrand N. Mukete, Dongtak Jeong, Shihong Zhang, Roger J. Hajjar, Djamel Lebeche

**Affiliations:** 1 Cardiovascular Research Institute, Icahn School of Medicine at Mount Sinai, New York, NY (I.K., A.H.C., S.K., B.N.M., D.J., S.Z., R.J.H.); 2 Graduate School of Biological Sciences, Mount Sinai School of Medicine, New York, NY (D.L.)

**Keywords:** gene therapy, hypertrophy/remodeling, left ventricular hypertrophy, left ventricular remodeling, microRNA

## Abstract

**Background:**

MicroRNAs (miRNAs) play a key role in the development of heart failure, and recent studies have shown that the muscle‐specific *miR‐1* is a key regulator of cardiac hypertrophy. We tested the hypothesis that chronic restoration of *miR‐1* gene expression in vivo will regress hypertrophy and protect against adverse cardiac remodeling induced by pressure overload.

**Methods and Results:**

Cardiac hypertrophy was induced by left ventricular pressure overload in male Sprague‐Dawley rats subjected to ascending aortic stenosis. When the hypertrophy was established at 2 weeks after surgery, the animals were randomized to receive either an adeno‐associated virus expressing *miR‐1* (AAV9.*miR‐1*) or green fluorescent protein (GFP) as control (AAV9.*GFP*) via a single‐bolus tail‐vein injection. Administration of *miR‐1* regressed cardiac hypertrophy (left ventricular posterior wall thickness,; 2.32±0.08 versus 2.75±0.07 mm, *P*<0.001) and (left ventricular septum wall thickness, 2.23±0.06 versus 2.54±0.10 mm, *P*<0.05) and halted the disease progression compared with control‐treated animals, as assessed by echocardiography (fractional shortening, 37.60±5.01% versus 70.68±2.93%, *P*<0.05) and hemodynamic analyses (end‐systolic pressure volume relationship/effective arterial elastance, 1.87±0.46 versus 0.96±0.38, *P*<0.05) after 7 weeks of treatment. Additionally, *miR‐1* replacement therapy lead to a marked reduction of myocardial fibrosis, an improvement in calcium handling, inhibition of apoptosis, and inactivation of the mitogen‐activated protein kinase signaling pathways, suggesting a favorable effect on preventing the maladaptive ventricular remodeling. We also identified and validated a novel bona fide target of *miR‐1*, Fibullin‐2 (*Fbln2*), a secreted protein implicated in extracellular matrix remodeling.

**Conclusions:**

Taken together, our findings suggest that restoration of *miR‐1* gene expression is a potential novel therapeutic strategy to reverse pressure‐induced cardiac hypertrophy and prevent maladaptive cardiac remodeling.

## Introduction

Cardiac hypertrophy is an adaptive growth response of the heart in response to increased stress such as aortic stenosis. Persistent stress activates diverse signal transduction pathways leading to pathological remodeling of the heart and consequently the development of heart failure, a syndrome characterized by dilatation of the left ventricle and contractile dysfunction.^1–3^ Cardiac hypertrophy is a major risk factor for the development of heart failure, and its therapeutic reversal is associated with improved mortality.^4–5^ Recent studies have indicated a key role of microRNAs (miRNAs) in biological processes including differentiation, apoptosis, proliferation, and development,^6–7^ and their dysregulation has been linked to several human diseases,^7^ including cardiovascular disease.^8–9^ miRNAs are small noncoding RNA molecules that act as post‐transcriptional repressors of target genes by antisense binding to 3′‐untranslated regions (3′‐UTRs) of target mRNAs, resulting in mRNA degradation and/or translational repression.^10^ Their altered expression has been linked to heart disease, and several miRNAs are aberrantly expressed in the diseased heart, suggesting that altered miRNA gene expression may be a common feature of human cardiovascular disease.^11–17^ Previous studies have shown that the muscle‐specific *miR‐1* is a key regulator of cardiac hypertrophy, and its expression is diminished in both animal models and human heart disease.^11–12,11–19^ Because individual miRNAs often regulate the expression of multiple gene targets, modulating the expression of a single miRNA can, in principle, influence multiple gene networks and thereby modify complex disease phenotypes. The network of *miR‐1* target genes and their mechanism of action in the heart have led to the suggestion that *miR‐1* gene replacement may provide a potential source of novel therapeutic targets for the treatment of cardiovascular abnormalities in humans.

In this study, we addressed a clinically relevant question of whether the chronic restoration of *miR‐1* expression in vivo by adeno‐associated virus (AAV)‐mediated gene transfer could be protective against the maladaptive cardiac remodeling induced by pressure overload. We present evidence that the restoration of *miR‐1* gene expression induces the regression of the pathological left ventricular (LV) pressure‐overload–induced hypertrophy and prevents the deterioration of cardiac function despite severe persistence of elevated LV systolic pressure. The reversal of the hypertrophic phenotype was also evident at the cellular and molecular levels and was paralleled by the attenuation of myocardial fibrosis and the inhibition of apoptosis. In addition, we identified a novel direct target gene of *miR‐1*,* Fbln2*, which, in part, may contribute to the regression of the hypertrophic phenotype. Our data reveal a novel mechanism of miR‐1–induced functional improvements that are associated with the modulation of hypertrophic signaling pathways, extracellular matrix (ECM) remodeling, and calcium homeostasis. These findings provide a proof‐of‐concept support for systemic delivery of antihypertrophic miRNAs using cardiotropic AAV vectors, which represent a novel strategy to modulate cardiac miRNA gene expression and potentially an attractive approach for heart failure therapy.

## Methods

Animals were handled as approved by the Mount Sinai Institutional Animal Care and Use Committee in accordance with the *Guidelines for the Care and Use of Laboratory Animals* published by the National Institutes of Health.

### Hypertrophy Model

Male Sprague‐Dawley rats (180 to 200 g) underwent ascending aortic banding (AAB) after induction of anesthesia with intraperitoneal ketamine (up to 85 mg/kg) and xylazine (up to 10 mg/kg), as described previously.^20^ Additional animals underwent a left thoracotomy without aortic banding to serve as age‐matched controls (sham).

### Recombinant AAV9.*miR‐1* Construct

A genomic fragment containing the *miR‐1* precursor was polymerase chain reaction (PCR) amplified from the rat *miR‐1* precursor sequence, cloned into self‐complementary AAV genome vector, and pseudotyped into rAAV9 capsids (AAV9.*miR‐1*). The green fluorescent protein (GFP)‐expressing vector (AAV9.GFP) was used as a control. Recombinant AAV‐9 viruses were produced as previously described.^21^

### Viral Delivery Protocol

**Figure 1.**
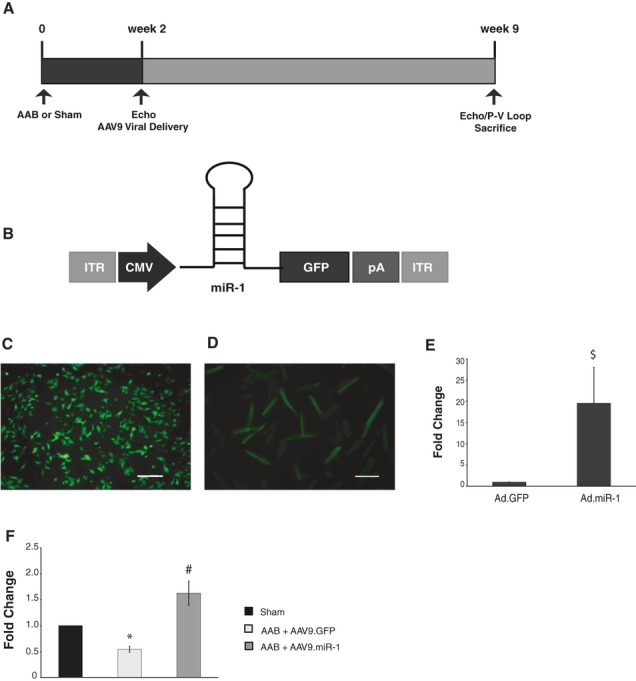
A, Overall design of the in vivo study. Rats were subjected to ascending aortic banding (AAB) or sham‐operated. Two weeks later, when hypertrophy was evident, the animals were randomly chosen to receive either adeno‐associated vector type 9 carrying *miR‐1* (AAV9.*miR‐1*) or control vector (AAV9.*GFP*) at 5×1011 vg (viral genomes) per animal via a single‐bolus tail‐vein injection. B, Schematic representation of *miR‐1* expression cassette. The expression cassette was comprised of the *miR‐1* stem‐loop sequence flanked by its native intron sequence, which preserves the putative hairpin structure and proper endogenous processing. A genomic fragment ≈800 bp containing the *miR‐1* precursor was PCR amplified from the rat *miR‐1* precursor sequence and cloned into the self‐complementary AAV9.*miR‐1* under the control of cytomegalovirus promoter. Infection of (C) rat neonatal or (D) rat adult ventricular cardiomyocytes with *miR‐1* expression virus in vitro, with (E) quantitative real‐time–polymerase chain reaction (qPCR) detection of mature *miR‐1* in rat neonatal myocytes 48 hours post infection in vitro (multiplicity of infection [MOI]=50) and (F) in vivo restoration of mature *miR‐1* expression evaluated as fold change relative to the sham‐operated animals at 7 weeks post gene transfer and assessed by real time qPCR. *miR‐1* expression levels were normalized to U6 rRNA. Values are mean±SE; sham: n=3; AAV9.*miR‐1*: n=6; AAV9.*GFP*: n=6 animals. Significance of differences: ^$^*P*<0.001, Ad.*miR‐1* vs Ad.*GFP*; ^#^*P*<0.05, AAV9.*miR‐1* versus AAV9.*GFP*; *P<0.05, AAV9.GFP versus sham.

Two weeks after AAB, the rats with evident LV hypertrophy, as assessed by echocardiography, were randomly chosen to receive a single‐bolus tail‐vein injection of either AAV9.*miR‐1* (n=6) or AAV9.GFP (n=9) at 5×10^11^ vg (viral genomes) per animal. Echocardiographic measurements were performed at baseline and 2 and 9 weeks post AAB. Invasive hemodynamics measurements were also obtained at 9 weeks post AAB, and the animals were killed (protocol summarized in Figure 1A).

### Echocardiographic Analysis

Transthoracic echocardiography was performed using a Vivid 7 (GE Healthcare) echocardiography apparatus with a 14‐MHz probe (i13L probe; General Electric). Animals were sedated with ketamine (up to 80 mg/kg) injected intraperitoneally. Long‐axis parasternal views and short‐axis parasternal 2‐dimensional (2D) views, at the mid‐papillary level, of the left ventricle were obtained to calculate the LV end‐diastolic (LVEDV) and end‐systolic (LVESV) volumes as well as the ejection fraction of the left ventricle. Volumes were calculated by using the formulae of the area‐length method (V=5/6×A×L, where V is the volume in mL, A is the cross‐sectional area of the LV cavity in cm^2^, obtained from the mid‐papillary short parasternal image in diastole and in systole, and L is the length of the LV cavity in cm, measured from the long‐axis parasternal image as the distance from the endocardial LV apex to the mitral‐aortic junction in diastole and in systole). M‐mode images were obtained by 2‐dimensional guidance from the parasternal short‐axis view for the measurements of LV wall thickness of the septum (cm) and of the posterior wall (cm), LV end‐diastolic diameter (cm), and LV end‐systolic diameter (cm), as well as to calculate the LV fractional shortening (FS, %).

### Hemodynamics Analysis

At end point, LV pressure–volume loops (P‐V) measurements were obtained as previously described.^22^ Briefly, rats were anesthetized with inhaled isoflurane (5% v/v) for induction and subsequently intubated and mechanically ventilated as noted in the surgery section. Isoflurane was lowered (2% to 3% v/v) for surgical incision. The chest was opened through a median sternotomy, and a 1.9F rat P‐V catheter (Scisense) was inserted into the LV apex through an apical stab performed with a 25‐gauge needle. The animals were kept sedated with 0.75% to 1% isoflurane maintaining a stable heart rate (≈350 beats/min). Hemodynamic recordings were performed after 5 minutes of stable heart rate. The intrathoracic inferior vena cava was transiently occluded to decrease venous return during the recording to obtain load‐independent P‐V relationships. Linear fits were obtained for end‐systolic P‐V relationships (ESPVR) and end‐diastolic P‐V relationships (EDPVR). At the end of the experiment, 30% NaCl 50 μL was slowly injected into the external jugular vein for ventricular parallel conductance measurement as previously described.^22–23^ Blood resistivity was measured using a special probe (Scisense). Volume measurements were initially obtained as blood conductance and calibrated using Baan equation 3, and pressure sensors were calibrated according to manufacturer's instructions (Scisense).

### Quantification of Mature miRNAs

Total RNA was isolated with use of an mirVana miRNA Isolation Kit (Ambion) followed by a DNase treatment to eliminate contaminating genomic DNA (Invitrogen). Mature *miR‐1* expression was quantified by real‐time quantitative PCR (qPCR) using the Taqman MicroRNA Assays according to the manufacturer's instructions (Applied Biosystems). Gene expression levels were normalized to U6 rRNA endogenous control, and fold changes were calculated using the ΔΔCt method.

### qRT‐PCR Gene Expression Analysis

Relative gene expression was determined using 2‐step qRT‐PCR. Quantitative PCRs were performed with Power SYBR Green Master Mix (Applied Biosystems) on an ABI Prism 7500 Real Time PCR System. Fold changes were calculated using the ΔΔCt method with normalization to 18S rRNA housekeeping gene.

### Western Blotting Analysis

Protein expression was evaluated in LV lysates by Western blot analysis according to standard procedures with antibodies against phospholamban (Pln) (Badrilla, UK), Serca2a (custom made in our laboratory), extracellular signal‐regulated protein kinase (ERK1/2), phospho‐ERK1/2 (Thr202/Tyr204), phospho‐p38 (Thr180/Tyr182), p38, phospho–c‐*jun* NH_2_‐terminal kinase (JNK) (Thr183/Tyr185), JNK, Bcl‐2, and Bax (Cell Signaling Technology), Fbln2 (Genetex), Ncx1 (Abcam), and Gapdh (Sigma‐Aldrich). The signals were detected with ECL‐Plus chemiluminescence detection kit (Pierce) or with the ODYSSEY Infrared Imaging System (Li‐CoR).

### Histological Assessment of Fibrosis

LV cryosections (≈10 μm) were fixed in 10% buffered formalin and stained with picrosirius solution (0.1% Sirius Red in picric acid; Sigma‐Aldrich). Images were acquired at ×20 magnification under circular polarized illumination using an Olympus BX50 microscope. The relative amount of collagen area to total tissue area was measured in each image by using a color threshold technique with National Institutes of Health ImageJ software.

### Histological Assessment of Myocyte Cross‐sectional Area

Heart LV cryosections (10 μm) cut at the papillary muscle level were fixed in 4% paraformaldehyde and incubated with Texas Red‐X conjugate of wheat germ agglutinin (5 μg/mL; Invitrogen) for 45 minutes at room temperature. The cross‐sectional area of approximately 100 myocytes with circular shape of the cell membrane was measured in the LV free walls of each animal using ImageJ software.

### Dual Luciferase Assays

The 3′‐UTR of Fbln2 and Ncx1 containing the predicted miR‐1 target sequences were amplified by PCR and cloned into the multiple cloning region located downstream of the *Renilla* translational stop codon of the psiCHECK‐2 dual luciferase vector (Promega). Using the DharmaFECT Duo transfection reagent (Thermo Fisher Scientific), 50 ng of psiCHECK–3′‐UTR constructs and 10 pmol of pre–*miR‐1* miRNA precursors or 10 pmol of pre‐miR negative control (Life Technologies) were cotransfected in HEK293FT (Invitrogen) cells. Forty‐eight hours after transfection, the normalized *Renilla* luciferase activity (*Renilla* luciferase/firefly) was measured using the Dual‐Glo Luciferase Assay System according to the manufacturer's instructions (Promega).

### Isolation and Culture of Rat Ventricular Myocytes

Neonatal rat ventricular myocytes were isolated by enzymatic dissociation of cardiac ventricle from 1‐ to 2‐day‐old Sprague‐Dawley pups using the neonatal cardiomyocyte isolation system according to the manufacturer's instructions (Worthington). Adult rat ventricular myocytes were isolated from male Sprague‐Dawley rats (280 to 300 g) using a modified Langendorff perfusion system as previously described.^24^

### Terminal Deoxynucleotidyl Transferase–Mediated dUTP Nick End Labeling Assay

The assessment of apoptosis was performed by the indirect terminal deoxynucleotidyl transferase–mediated dUTP nick end labeling assay (TUNEL) method, using an antidigoxigenin antibody with a rhodamine fluorochrome with the Apoptag Red in Situ Apoptosis Detection kit (Millipore) according to the manufacturer's protocol.

### Human Heart Specimens

Human heart tissue specimens were obtained from the National Disease Research Interchange through the Human Tissues and Organs for Research program.

### Statistical Analysis

The underlying assumption of normal distribution was investigated by performing a Kolmogorov–Smirnoff normality test and normal probability plot test. When the distribution was found to be normal, statistical significance between 2 groups was examined by *t* test and by 1‐way ANOVA for multigroup comparisons. When the ANOVA results were significant, the differences among individual groups were determined with the Bonferroni post hoc test. The echocardiography data were analyzed with repeated measures ANOVA test. The luciferase reporter assay data were analyzed with 2‐factor ANOVA test. *P*<0.05 was considered significant.

## Results

### Systemic Administration of AAV9.*miR‐1* Restores *miR‐1* Gene Expression In Vivo

A genomic fragment containing the *miR‐1* precursor was PCR amplified from the rat *miR‐1* precursor sequence and cloned into the self‐complementary AAV genome vector under the control of cytomegalovirus promoter (Figure 1B). The expression cassette was comprised of the *miR‐1* stem loop sequence flanked by its native intron sequence, which preserves the putative hairpin structure and proper endogenous processing. The expression of mature *miR‐1* was validated in vitro in neonatal (Figure 1C) and adult cardiac myocytes (Figure 1D) by qRT‐PCR with stem‐loop primers (Figure 1E). AAV9‐mediated *miR‐1* gene transfer in vivo in the setting of pressure‐overload hypertrophy (Figure 1A) restored the expression of the mature *miR‐1* in the hypertrophic hearts (Figure 1F).

### *miR‐1* Gene Transfer Reverses Hypertrophy and Prevents Functional Deterioration

**Figure 2.**
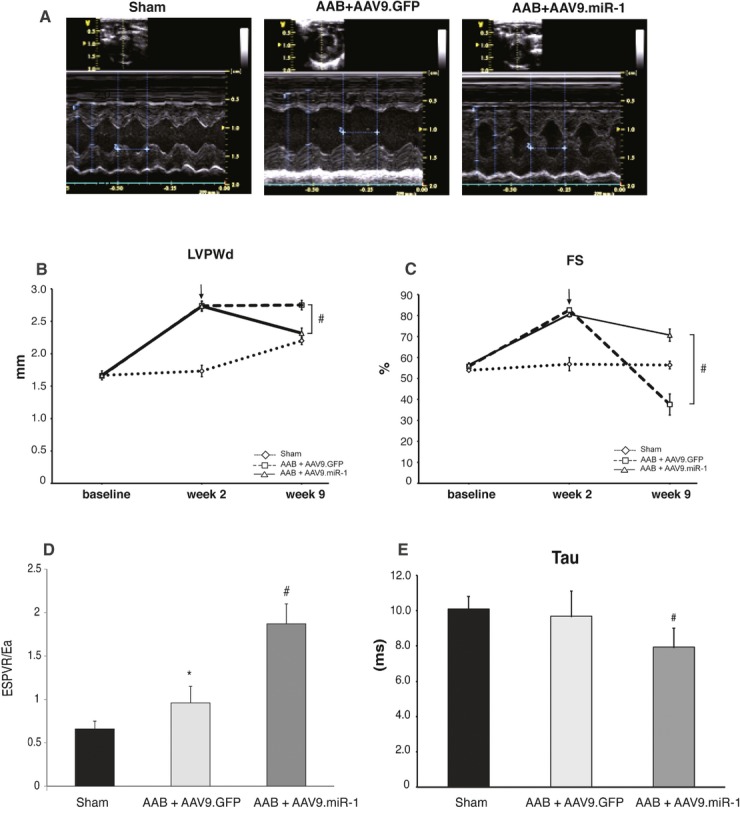
Effects of *miR‐1* gene transfer on cardiac function in vivo. A, Representative raw tracings of M‐mode echocardiography 7 weeks after gene delivery. B, Echocardiographic measurements of left ventricular posterior wall dimensions (LVPWd; mm) and (C) LV fractional shortening (FS, %) at baseline and 2 weeks and 9 weeks post ascending aortic banding (AAB); arrow in (B) and (C) indicates the time of adeno‐associated vector type 9 (AAV9) delivery mean±SE, sham: n=3; AAV9.*miR‐1*: n=6; AAV9.*GFP*: n=9 animals. D, In vivo hemodynamics analyses of the ratio of the end‐systolic pressure–volume relationship over the effective arterial elastance (ESPVR/Ea), an index of left ventricular efficiency; and (E) the time constant of left ventricular pressure decay during the isovolumic relaxation phase (Tau) (mean±SE, sham: n=3; AAV9.*miR‐1*: n=4; AAV9.GFP: n=4 animals). Significance of differences: *P<0.05, AAV9. GFP vs sham; ^#^*P*<0.05, AAV9.*miR‐1* vs AAV9.*GFP*.

LV function and dimensions were measured by serial echocardiography at baseline and 2 weeks and 9 weeks post‐AAB (Figure 2A). After 2 weeks, the AAB hearts exhibited concentric hypertrophy characterized by marked increase in LV thickness (LV wall thickness of the septum and of the posterior wall) and a significant increase in fractional shortening (FS) compared with sham‐operated animals (Table 1). Seven weeks after gene transfer, the LV wall thickness of the AAV9.*miR‐1* group was significantly decreased compared with the AAV9.GFP group (LV wall thickness of the posterior wall: 2.32±0.08 versus 2.75±0.07 mm, *P*<0.001; LV wall thickness of the septum: 2.23±0.06 versus 2.54±0.10 mm, *P*<0.05) (Figure 2B and Table 1). In addition, the AAV9.GFP group displayed substantial increase in LV chamber end‐systolic (LVEDs) and end‐diastolic (LVEDd) dimensions compared with the sham group, indicating ventricular dilatation, which has been significantly attenuated by *AAV9.miR‐1* gene transfer (Table 1). The AAV9.*GFP* group exhibited significantly reduced contractility compared with the AAV9.*miR‐1*–treated animals, as assessed by FS (37.60±5.01% versus 70.68±2.93%, *P*<0.05) (Figure 2C) and the ejection fraction (53.86±7.12% versus 87.99±1.43%, *P*<0.05) (Table 1).

**Table 1.** LV Function and Dimensions Were Measured by Serial Echocardiography at Baseline, 2 and 9 Weeks Post‐AAB

	IVSd, mm	LVPWd, mm	LVIDd, mm	LVIDs, mm	FS, %	EF, %	EDV, μL	ESV, μL
Baseline								
Sham	1.67±0.03	1.66±0.03	5.97±0.03	2.73±0.03	53.88±0.67	80.93±1.93	341.69±7.31	65.33±7.35
AAV9.GFP	1.68±0.06	1.66±0.03	6.05±0.09	2.68±0.10	55.79±1.43	83.17±1.13	335.77±4.89	58.36±3.07
AAV9.*miR‐1*	1.65±0.08	1.67±0.07	5.87±0.17	2.58±0.09	56.45±1.02	82.59±1.15	339.90±2.19	57.08±4.46
Week 2								
Sham	1.73±0.03	1.73±0.09	7.03±0.32	3.03±0.28	56.81±3.14	81.45±1.55	422.11±11.31	77.63±5.70
AAV9.GFP	2.47±0.10*	2.74±0.07*	5.81±0.13*	0.97±0.06*	82.62±0.89*	93.24±0.11*	328.51±4.04*	22.23±0.50*
AAV9.*miR‐1*	2.47±0.12*	2.73±0.08*	5.30±0.34*	1.02±0.06*	80.58±1.25*	93.30±0.31*	340.06±10.14*	22.97±1.45*
Week 9								
Sham	2.10±0.06	2.20±0.06	7.20±0.10	3.13±0.15	56.40±1.82	73.48±5.76	601.56±22.16	160.69±36.62
AAV9.GFP	2.54±0.10*	2.75±0.07*	8.32±0.45	5.35±0.66*	37.60±5.01*	53.86±7.12*	882.46±41.4*	429.38±80.21*
AAV9*.miR‐1*	2.23±0.06*	2.32±0.08*	7.18±0.19*	2.10±0.17*^,^*	70.68±2.93*^,^*	87.99±1.43*^,^*	530.06±28.94*	65.10±10.79*^,^*

Data are mean±SE; sham: n=3; AAV9.*GFP*: n=9; AAV9.*miR‐1*: n=6.LV indicates left ventricular; AAV9.*miR‐1*, adeno‐associated virus expressing *miR‐1*; IVSd, intraventricular septum thickness in diastole; LVPWd, left ventricular posterior wall thickness in diastole; LVIDd/s, LV end‐diastolic/systolic diameter; AAB, ascending aortic banding; FS, fractional shortening; EF, ejection fraction; EDV, end‐diastolic volume; ESV, end‐systolic volume; GFP, green fluorescent protein.

* *P*<0.05, AAV9.GFP vs sham.

* *P*<0.01, AAV9.*miR‐1* vs AAV9.*GFP*.

* *P*<0.05, AAV9.*miR‐1* vs sham.

Additionally, we further examined the effects of *miR‐1* restoration on cardiac function in vivo by using the PV‐loop analysis. Catheterization and hemodynamic analysis after 7 weeks of gene transfer showed a significant increase in LV chamber dimensions in the AAV9.*GFP*– compared with the AAV9.*miR‐1*–treated hearts (EDV: 1316.30±79.15 μL versus 683.37±79.14 μL, *P*<0.05; ESV: 869.40±150 μL versus 312.86±83.85 μL, *P*<0.05) (Table 2), which is in agreement with the echocardiographic measurements. The analysis of LV function also revealed a significant improvement in the AAV9.*miR‐1* systolic parameters compared with the AAV9.GFP group as measured by the end‐systolic PV relation normalized to the effective arterial elastance (Ea) (1.87±0.46 versus 0.96±0.38, *P*<0.05) (Figure 2D). Moreover, diastolic parameters were also normalized in the AAV9.*miR‐1* group as evidenced by the decrease in the time constant of LV pressure decay during the isovolumic relaxation phase tau (7.94±0.7 versus 9.69±1.42, *P*<0.05) (Figure 2E). Taken together, these data suggest that *miR‐1* gene transfer preserved cardiac function and prevented the transition to heart failure induced by pressure overload.

**Table 2.** LV Hemodynamic Measurements by Pressure–Volume Conductance Catheters at 9 Weeks Post Banding

	Sham (n=3)	AAB+AAV9.*GFP* (n=4)	AAB+AAV9.*miR‐1* (n=4)
Tau, ms	10.10±0.62	9.69±1.42	7.94±0.70*
LVEDV, μL	794.11±21.51	1316.3±79.15*	683.37±79.14*
LVESV, μL	379.26±29.13	869.40±150*	312.86±83.85*
EF, %	52.00±0.87	35.98±8.0*	56.85±3.5*
ESPVR/Ea	0.66±0.09	0.96±0.19	1.87±0.23*

Data represents mean±SE. LV indicates left ventricular; AAB, ascending aortic banding; AAV9.*miR‐1*, adeno‐associated virus expressing *miR‐1*; LVEDV, left ventricular end‐diastolic volume; LVESV, left ventricular end‐systolic volume; EF, ejection fraction; ESPVR, end‐systolic pressure–volume relationship; Ea, arterial elastance.

* *P*<0.05, AAV9.*GFP* vs sham.

* *P*<0.05, AAV9.*miR‐1* vs AAV9.*GFP*.

**Figure 3.**
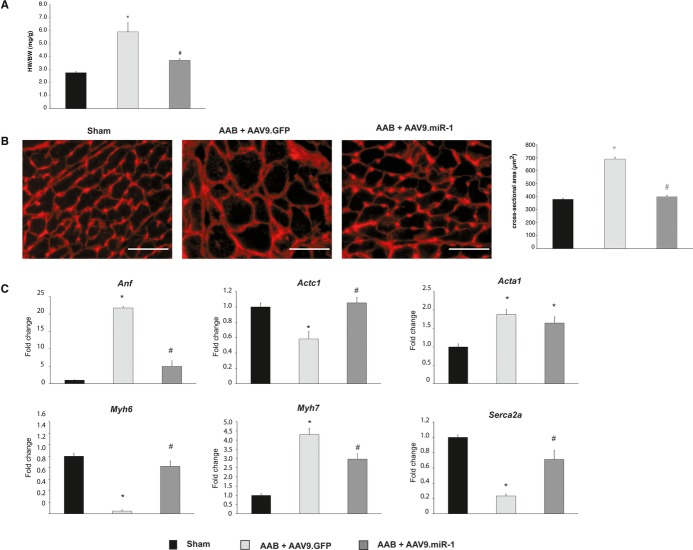
Assessment of the cardiac hypertrophy at the cellular and molecular level. A, Ratio of heart weight (HW, mg) to body weight (BW, g) at 7‐week post gene transfer (mean±SE of n=6 animals per group). Significance of differences: *P<0.01 AAV9.*GFP* vs sham, ^#^*P*<0.01 AAV9.*miR‐1* vs AAV9.*GFP*. B, Representative epifluorescence images of left ventricular histological sections stained with Texas‐Red conjugated wheat germ agglutinin (WGA‐Texas Red). Scale bar: 50 μm, cardiomyocyte cross‐sectional area (μm^2^) measurements (mean±SE measured from n=300 cardiomyocytes). Significance of differences: *P<0.05, AAV9.*GFP* vs sham; ^#^*P*<0.05, AAV9.*miR‐1* vs AAV9.*GFP*. C, The expression levels of the cardiac genes *Anf*,* Acta1*,* Actc1*,* Myh7*,* Myh6*, and *Serca2a* were evaluated as fold change relative to the sham‐operated animals at 7 weeks post gene transfer (mean±SE of a minimum n=3 animals per group). Values represent mean±SE; sham: n=3; AAV9.*miR‐1*: n=6; AAV9.GFP: n=6 animals. Significance of differences: *P<0.05, AAV9.GFP vs sham, ^#^*P*<0.05, AAV9.*miR‐1* vs AAV9.*GFP*. AAV9 indicates adeno‐associated vector type 9; AAB, ascending aortic banding; *Anf*, atrial natriuretic factor; *Acta1*, skeletal muscle α‐actin; *Actc1*, cardiac α‐actin; *Myh6*, alpha‐myosin heavy chain; *Myh7*, beta‐myosin heavy chain.

Postmortem analysis revealed that the heart weight–to–body weight ratio was significantly lower in AAV9.*miR‐1–* compared with AAV9.GFP–treated hearts (3.72±0.10 versus 5.9±0.69 mg/g, *P*=0.02) (Figure 3A). We confirmed these observations at the cellular level by evaluating the cross‐sectional area of cardiac myocytes in histological sections of LV tissues at 7 weeks post gene transfer (Figure 3B). We observed that cardiac myocytes from AAV9.GFP were significantly larger than those from the sham‐operated hearts (689.33±14.53 versus 379.68±9.10 μm^2^, *P*<0.05). In contrast, AAV9.*miR‐1* treatment significantly reduced the cardiac myocyte cross‐sectional area to levels similar to those of the sham‐operated hearts (400.92±12.62 versus 379.68±9.10 μm^2^, *P*<0.05) (Figure 3B).

### *miR‐1* Modulates the Expression of Molecular Markers of Cardiac Hypertrophy

Next we investigated the effect of *miR‐1* treatment on molecular abnormalities associated with pathological hypertrophy. We tested the hypothesis that the reversal of the pathological remodeling observed in the AAV9.*miR‐1* group in vivo is paralleled by a reversal in the reinduction of the maladaptive fetal cardiac gene program. We assessed the expression of the hypertrophic fetal genes, atrial natriuretic factor (*Anf*), skeletal muscle α‐actin (*Acta1*), cardiac alpha‐actinin (*Actc1*), as well as the contractile protein isoforms α‐ and β‐myosin heavy chain (*Myh6* and *Myh7*, respectively), at 7 weeks post gene transfer. Pressure‐overload–induced hypertrophy in the AAV9.*GFP*–treated group was associated with reinduction of the “fetal gene program” characterized by a significant increase in the mRNA expression of *Anf* (21‐fold), *Myh7* (4.3‐fold), and *Acta1* (1.8‐fold) and a decrease in *Myh6* (21.6‐fold) and *Actc1* (1.7‐fold) compared with sham‐operated animals (Figure 3C). In contrast, we observed a reversal of the MHC shift that was characterized by the significant increase in the “adult” *Myh6* (17.84‐fold) and decrease in the “fetal” *Myh7* (1.55‐fold) as well as the significant decrease in *Anf* expression (4.37‐fold) and the significant increase of *Actc1* (1.8‐fold) in miR‐1‐treated hearts compared with AAV9.*GFP*–treated hearts (Figure 3C).

### *miR‐1* Regulates the Expression and Activity of Key Calcium Homeostasis Genes

**Figure 4.**
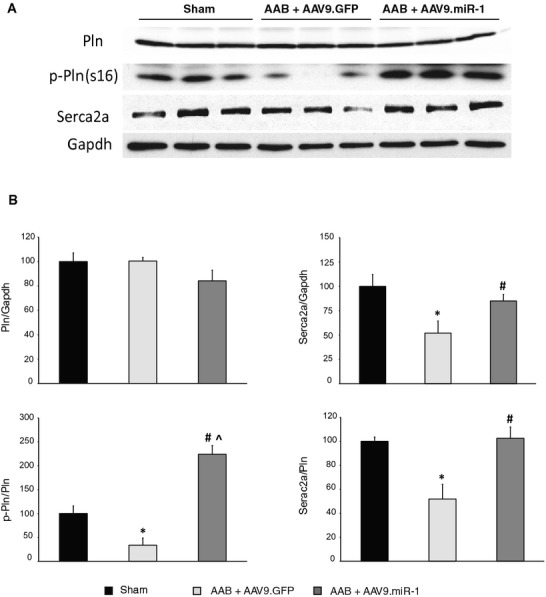
Expression analysis of key calcium cycling proteins. A, Representative blots of the protein expression levels of *Pln*, phospho‐*Pln* and *Serca2a*. B, Densitometric analysis of protein levels *Pln*, phospho‐*Pln* and *Serca2a*, evaluated as percentage change over that in sham‐operated animals (100%) (mean±SE; sham: n=3; AAV9.*miR‐1*: n=6; AAV9.GFP: n=6 animals.). *Gapdh* protein expression was used as loading control. *P<0.05 AAV9.GFP versus sham; ^#^*P*<0.05, AAV9.*miR‐1* vs AAV9.GFP; ^*P*<0.05, AAV9.*miR‐1* vs sham. AAV9 indicates adeno‐associated vector type 9; AAB, ascending aortic banding; *Pln*, phospholamban; *Serca2a*, sarcoplasmic reticulum Ca^2+^‐ATPase.

Ca^2+^ uptake into the sarcoplasmic reticulum (SR) is mediated by the cardiac SR Ca^2+^‐ATPase (*Serca2a*) and regulated by *Pln*.^25^ *Serca2a* is an important regulator of intracellular Ca^2+^ signaling in the heart, and its decreased expression and activity are implicated in heart failure.^26^ We performed immunoblotting analyses to assess the protein levels of *Serca2a* and *Pln* and the phosphorylation state of *Pln* (Figure 4A). We observed a significant decrease in *Serca2a* protein expression levels (50%) but no change in *Pln* expression in ventricular tissue from AAV9.*GFP* compared with sham‐operated animals (Figure 4B). Phosphorylation of *Pln* at Ser16 was also decreased (Figure 4B). In contrast, AAV9.*miR‐1* significantly upregulated *Serca2a* protein levels and restored the *Serca2a/Pln* ratio to sham levels compared with AAV9.GFP; it also increased *Pln* phosphorylation at Ser16 and the phospho*Pln/Pln* ratio (Figure 4B). Taken together, the data suggest that the changes in *Serca2a* content and activity (increased *Pln* phosphorylation and *Serca2a/Pln* ratio) are indicative of enhanced SR Ca^2+^ cycling, which correlates with the improvement in cardiac contractility observed following *miR‐1* gene transfer.

### Activity of the Mitogen‐Activated Protein Kinase Signaling Pathways Is Altered by *miR‐1*

**Figure 5.**
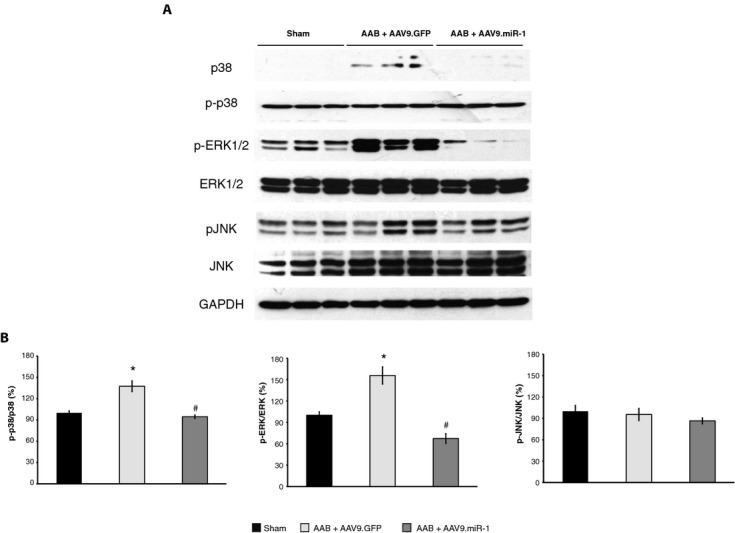
MAPK signaling pathway analysis. A, Representative blots of phosphorylated and total protein levels of ERK1/2, p38, and JNK from heart of sham‐operated animals and AAB animals treated with either AAV9.*GFP* or AAV9.*miR‐1*. B, Densitometric analysis of phosphorylated to total protein levels evaluated as percentage change over that in sham‐operated animals (100%) (mean±SE; sham: n=3; AAV9.*miR‐1*: n=6; AAV9.*GFP*: n=6 animals.). *Gapdh* protein expression was used as loading control. *P<0.05, AAV9.GFP vs sham; ^#^*P*<0.01, AAV9.*miR‐1* vs AAV9.*GFP*. AAV9 indicates adeno‐associated vector type 9; AAB, ascending aortic banding; ERK, extracellular signal‐regulated protein kinase; JNK, c‐*jun* NH_2_‐terminal kinase.

It is well established that pressure‐overload–induced biomechanical stresses stimulate various signaling pathways essential for induction of the hypertrophic response.^3^ These signaling pathways include the mitogen‐activated protein kinase superfamily with its 3 terminal effector kinase subfamilies: ERK1/2, JNK, and p38.^27^ We sought to determine whether *miR‐1* restoration had an effect on these pathways. The activity of both ERK1/2 and p38 kinase proteins were significantly increased in AAV9.*GFP* hearts compared with sham‐operated animals, as assessed by their phosphorylation levels. In contrast, the p38 and the ERK1/2 kinase activation was significantly decreased in AAV9.*miR‐1–*compared with AAV9.*GFP* treated animals. We did not observe significant changes in JNK or phospho‐JNK levels among the groups (Figure 5).

### *miR‐1* Inhibits Cardiac Fibrosis and Apoptosis

**Figure 6.**
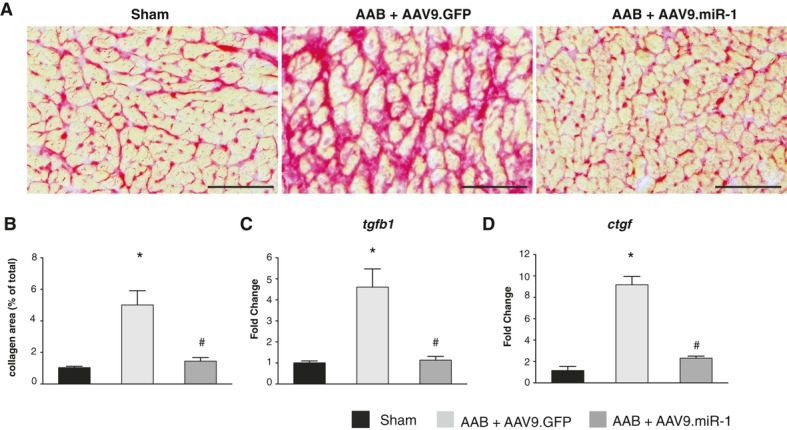
Assessment of myocardial fibrosis. A, Representative micrographs of Sirius Red–stained cryosections of LV myocardium from animals treated as indicated. B, Quantification of the myocardial collagen fraction. Relative mRNA expression levels of the (C) *Tgfb1* and (D) *Ctgf* genes, normalized to 18S expression, were evaluated as fold induction over that of the sham‐operated animals at 7 weeks post gene transfer (mean±SE; sham: n=3; AAV9.*miR‐1*: n=6; AAV9.GFP: n=6 animals.). Significance of differences: *P<0.05, AAV9.GFP vs sham; ^#^*P*<0.05, AAV9.miR‐1 vs AAV9.GFP. Scale bars: 100 μm. LV indicates left ventricular; AAV9, adeno‐associated vector type 9; AAB, ascending aortic banding; *Tgfb1*, transforming growth factor β‐1; *Ctgf*, connective tissue growth factor.

Since cardiac fibrosis and apoptosis are prominent features in the transition from compensatory hypertrophy to heart failure, we sought to examine the potential involvement of *miR‐1* restoration in the regulation of cardiac ECM remodeling and apoptosis. Fibrosis is a pathological feature of cardiac adaptation to stress, where the proliferation of fibroblasts and increased deposition of ECM components results in myocardial stiffness and diastolic dysfunction,^28^ and recently it has been demonstrated that miRNAs play a central role in the control of cardiac fibrosis and pathological LV remodeling.^29–31^ Histological examination of LV sections by sirius‐red staining and subsequent quantification of the fibrotic area revealed that AAB induced a profound increase in interstitial fibrosis in the AAV9.GFP–treated hearts compared with sham‐operated hearts. In contrast, AAV9.*miR‐1* treatment significantly decreased fibrosis (Figure 6A and 6B). In addition, we observed a significant reduction in mRNA expression of the profibrotic genes, the transforming growth factor beta‐1 (*Tgfb1*), and the connective tissue growth factor (*Ctgf*), in the AAV9.*miR‐1–* compared with AAV9.GFP–treated hearts (Figure 6C and 6D, respectively).

**Figure 7.**
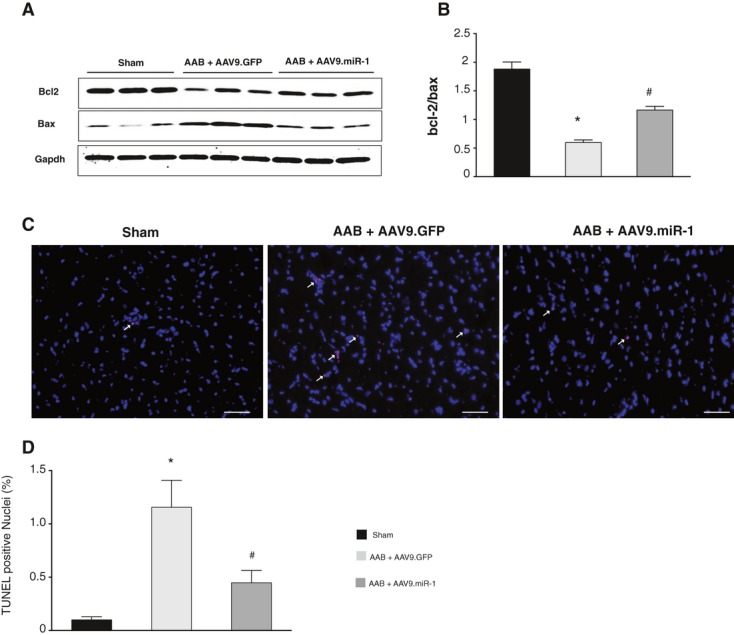
Assessment of apoptosis. A, Western blot analysis of bcl‐2 and bax protein expression. *Gapdh* protein expression was used as loading control. B, Densitometric analysis of the bcl‐2/bax ratio. Values represent mean±SE; sham: n=3; AAV9.*miR‐1*: n=6; AAV9.GFP: n=6 animals. C, Representative epifluorescence images of TUNEL labeling of LV histological sections at 7 weeks post gene transfer. Arrows indicate TUNEL‐positive nuclei. D, Quantification of TUNEL‐positive nuclei. Values are mean±SE measured from 10 000 nuclei. *P<0.05, AAV9.GFP vs sham; ^#^*P*<0.05, AAV9.*miR‐1* vs AAV9.GFP. Scale: 50 μm. LV indicates left ventricular; AAV9, adeno‐associated vector type 9; AAB, ascending aortic banding; TUNEL, terminal deoxynucleotidyl transferase–mediated dUTP nick end labeling assay.

In addition, in response to long‐term pressure overload, cardiomyocyte apoptosis may further contribute to the transition from LV hypertrophy to heart failure.^32^ By Western blot analysis, we quantified the protein expression of the antiapoptotic gene, Bcl‐2, and the proapoptotic gene, Bax, which are involved in the apoptotic pathway (Figure 7A). Results showed that there was a significant increase in Bcl‐2 and a decrease in Bax expression in AAV9.*miR‐1*– compared with AAV9.*GFP*–treated hearts. Consequently, the Bcl‐2/Bax ratio, an important marker of myocardial cell survival probability,^32^ was significantly increased in the AAV9.*miR‐1*– compared with AAV9.*GFP*–treated hearts (Figure 7B). These findings were further corroborated in situ apoptosis detection by TUNEL assay, which demonstrated a marked decrease in DNA fragmentation of nuclei detected in AAV9.*miR‐1*– compared with AAV9.*GFP*–treated hearts (Figure 7C through 7D).

### Identification of *Fbln2* as a Direct Target of *miR‐1*

**Figure 8.**
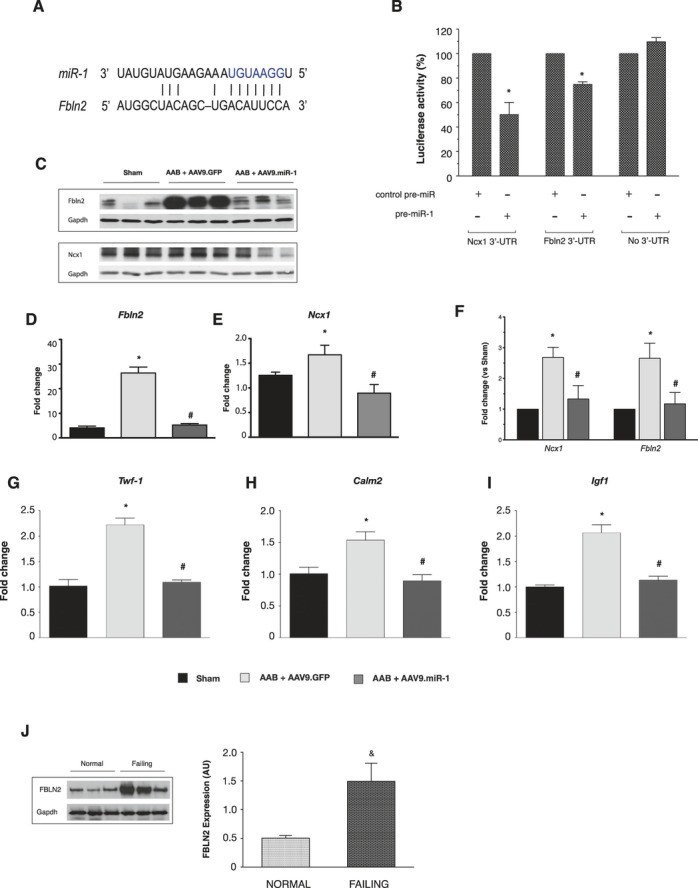
Identification and validation of the *miR‐1* direct target genes. A, Bioinformatics analysis identified *Fbln2* as a putative target gene of *miR‐1*. The mRNA sequence of *Fbln2* is predicted to contain a conserved “seed” sequence complimentary to *miR‐1* in the 3′‐untranslated region (3′‐UTR). B, Luciferase reporter assays performed by cotransfection of miR‐1 “mimics” (pre–*miR‐1*) with a luciferase vector linked to the *Fbln2* or *Ncx1* 3′‐UTR or deleted 3′‐UTR complementary site (No 3′‐UTR); a control nontargeting “mimic” (pre–miR‐control) was also included. Data represent mean±SE of n=6 experiments per condition, ^$^*P*<0.001. C, Representative Western blots of Fbln2 and *Ncx1* protein expression. D, Relative densitometric analysis of *Fbln2* protein expression and (E) *Ncx1* protein expression. Data represent mean±SE; sham: n=3; AAV9.*miR‐1*: n=6; AAV9.GFP: n=6 animals. F, The relative mRNA gene expression levels of *Fbln2* and *Ncx1*, normalized to 18S expression, were evaluated as fold induction over that in the sham‐operated animals at 7 weeks post gene transfer. g and h, Gene expression analysis of previously identified *miR‐1* target genes. The expression levels of the target genes *Twf‐1* (G), *Calm2* (H), and *Igf1* (I) were evaluated as fold change relative to the sham‐operated animals at 7 weeks post gene transfer. Expression levels were normalized to 18S expression. Values represent mean±SE; sham: n=3; AAV9.*miR‐1*: n=6; AAV9.GFP: n=6 animals. Significance of differences: *P<0.05, AAV9.*GFP* vs sham, ^#^*P*<0.05, AAV9.*miR‐1* vs AAV9.*GFP*. J, Assessment of *FBLN2* expression in human heart samples. Representative blots of the protein levels of *FBLN2* in both normal and failing human heart samples and densitometric analysis of the protein expression levels (mean±SE of n=3 samples per group). *Gapdh* protein expression was used as loading control. ^$^*P*<0.05 failing vs normal. AAV9 indicates adeno‐associated vector type 9; AAB, ascending aortic banding; *Twf‐1*, twinfilin 1; *Calm2*, calmodulin 2; *Igf1*, insulin growth factor 1; *Fbln2*, fibullin‐2

Previous studies have identified several direct targets of miR‐1, including calmodulin (*Calm*),^18^ insulin growth factor 1 (*Igf1*),^19^ *Ncx1*,^33^ and twinfilin (*Twf1*),^34^ which play a significant role in hypertrophy and heart failure. To gain additional insights into the molecular function of the *miR‐1* in the setting of pressure‐overload–induced cardiac hypertrophy, we used computational and experimental approaches for the identification of novel, biologically relevant direct target genes. Using a target prediction algorithm,^35^ we identified *Fbln2 as* putative target gene of *miR‐1*. The mRNA sequence of *Fbln2* is predicted to contain a conserved “seed” sequence complementary to *miR‐1* in the 3′‐UTR (Figure 8A). *Fbln2* is an extracellular matrix protein that is highly expressed in the developing heart^36^ and plays an important role during embryonic development^37^ and cardiac remodeling.^38^

We performed a luciferase‐based expression assays in vitro to verify that *Fbln2* is a bona fide *miR‐1* target. A short (≈250 bp) fragment from the 3′‐UTR of *Fbln2* was cloned downstream of the stop codon of the *Renilla* luciferase in a dual‐luciferase reporter vector. In parallel, a fragment of the *Ncx1* 3′‐UTRs predicted to contain a conserved “seed” sequence complementary to *miR‐1* was also cloned, serving a positive control for the luciferase assay. Each construct was cotransfected in HEK293 cells with synthetic *miR‐1* mimics (pre–*miR‐1*) or control mimics (control pre‐miR). In the presence of *miR‐1,* we observed a significant decrease in luciferase activity of *Fbln2* and *Ncx1* constructs (Figure 8B). In contrast, cotransfection of a control miRNA did not result in a decrease in luciferase activity (Figure 8B). In addition, cotransfection of pre‐*miR‐1* or control pre‐miR mimics with constructs containing the deleted 3′‐UTR sequences had no significant effect on the luciferase activity (Figure 8B).

In addition, we evaluated whether *Fbln2* and *Ncx1* expression was regulated by *miR‐1* in vivo in the setting of pressure‐overload–induced hypertrophy. Western blot analysis demonstrated that at the protein level, the expression of both *Fbln2* and *Ncx1* was significantly decreased in the LV tissue of the AAV9.*miR‐1*– compared with AAV9.*GFP*–treated animals (Figure 8C through 8E). In addition to translational repression, miRNAs can lead to mRNA degradation of their targets.^39–40^ We observed a significant decrease in the mRNA expression levels of *Fbln2* and *Ncx1* in the AAV9.*miR1* animals compared with AAV9.*GFP*‐treated, as assessed by qRT‐PCR (Figure 8F). Furthermore, we verified that the expression of *Igf1*,* Twf1*, and *Calm2* was decreased in AAV9.*miR‐1*– compared with AAV.9.*GFP*–treated animals (Figure 8G through 8I). Taken together, these results suggest that the restoration of *miR‐1* levels in the setting of pressure‐overload–induced hypertrophy in vivo was paralleled by the modulation of a novel direct target gene, *Fbln2,* as well as the previously identified targets, *Ncx1*,* Igf1*,* Twf1*, and *Calm2*.

## Discussion

Recent studies have shown that changes in miRNA expression play an important role in diverse aspects of cardiovascular pathophysiology, and the modulation of miRNA activity could provide potential new therapeutic targets for cardiovascular diseases.^9,41^ The ability of a single miRNA to control the expression of hundreds of proteins^42–43^ suggests that modulation of individual miRNAs can influence multiple pathways simultaneously. Thus, therapeutically restoring the levels of individual antihypertrophic miRNAs in the hypertrophic heart, such as *miR‐1*, may therefore alleviate the deleterious effect of multiple pathways associated with pathological ventricular remodeling and heart failure.

In the current study, we assessed the long‐term effect of *miR‐1* gene transfer in pressure‐overload–induced cardiac hypertrophy in vivo using a cardiotropic AAV9 vector that efficiently transduces cardiac tissue.^21,44–45^ We have presented a novel therapeutic strategy for the treatment of preexisting hypertrophy based on cardiac‐targeted delivery of *miR‐1*. The results described herein demonstrated that normalization of *miR‐1* gene expression levels, which were downregulated in hypertrophy, reversed cardiac hypertrophy and attenuated pathological remodeling by simultaneously affecting multiple processes associated with pathological hypertrophy and heart failure. These findings are in agreement with other data showing an antihypertrophic effect of *miR‐1*.^11–12,18^ Further support to our findings is provided by recent studies that showed that *miR‐1* overexpression attenuated agonist‐induced cardiomyocyte hypertrophy both in vitro^11–12^ and in vivo.^18^ However, in the latter study of Ikeda et al,^18^ only the short‐term expression of *miR‐1* was examined in normal hearts using an adenovirus.

An important and novel aspect of our study is the uncovering of the pivotal role that *miR‐1* plays in the attenuation of pressure‐overload–induced myocardial fibrosis, a key pathological feature of myocardial remodeling. Chronic pressure overload induces structural changes characterized by an increased accumulation of ECM proteins in the interstitium and perivascular regions of the myocardium, which leads to increased myocardial stiffness and alters the mechanics of the heart, predisposing individuals to ventricular dysfunction and arrhythmias.^28,46–47^ However, the molecular mechanisms underlying the development of a fibrogenic cardiac phenotype are not yet fully defined. Recently, it has been demonstrated that miRNAs such as *miR‐133*,* miR‐30*,* miR‐29*, and *miR‐21* play a central role in the control of cardiac fibrosis and pathological LV remodeling by targeting multiple ECM‐related proteins associated with fibrosis.^29–31^ In line with these findings, our data suggest that *miR‐1* may also be implicated in the regulation of fibrosis by targeting Fbln‐2, a secreted ECM protein that plays an important role during adverse tissue remodeling under pathologicsl conditions.^38,48–49^ It has been recently described that the loss of Fbln2 inhibits ECM remodeling, attenuating the progression of cardiac remodeling after myocardial infarction.^38^ Consistent with the postulated role of *Fbln2* as a key pro fibrotic factor, we showed that the restoration of *miR‐1* expression significantly decreased its expression as well as the marked reduction in collagen content and the downregulation of key profibrotic factors. The significance of this observation is strengthened by data from human samples showing that *Fbln2* protein expression is significantly increased in hypertrophic hearts (Figure 8J), suggesting that it may be a key player in the development of heart failure in humans.

**Figure 9.**
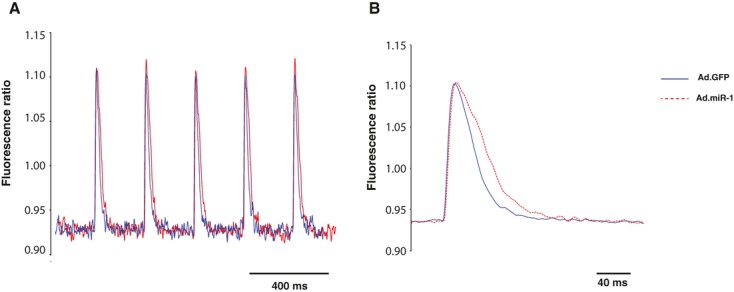
[Ca^2+^]_i_ transients in isolated hypertrophic adult rat ventricular cardiomyocytes (ARVMs). Cardiomyocytes were isolated for pressure‐overload–induced hypertrophic hearts at 3 weeks post ascending aortic banding and infected with an adenovirus expressing either *miR‐1* (Ad.*miR‐1*) or a control adenovirus (Ad.*GFP*). After 48 hours, the cardiomyocytes were loaded with a fluorescent Ca^2+^‐sensitive dye, Fura‐2, and the ratio of the fluorescence intensities (excited at 340 and 380 nm) were recorded using the IonOptix system. A, Representative ([Ca^2+^]_i_) transient raw traces of individual cardiomyocytes and (B) averaged traces used for transient analysis.

We have also uncovered another potential novel role of *miR‐1* in intracellular Ca^2+^ homeostasis. To maintain Ca^2+^ homeostasis, Ca^2+^ entering cardiac cells during the process of excitation‐contraction coupling must be balanced by Ca^2+^ removal. During relaxation, Ca^2+^ is immediately transported into the SR via *Serca2a* and extruded by the sarcolemmal *Ncx1*.^50^ Heart failure is associated with reduced *Serca2a* expression^51–53^ and increased *Ncx1* expression,^54–56^ which has been shown to contribute to both contractile dysfunction and arrhythmogenesis.^54^ Our data suggests a beneficial effect of *miR‐1* gene transfer in Ca^2+^ homeostasis by directly modulating *Ncx1* expression levels. In subsequent experiments in isolated hypertrophic cardiomyocytes, we demonstrated that *miR‐1* overexpression modulates intracellular Ca^2+^ transients by altering the kinetics of Ca^2+^ removal from the cytoplasm during relaxation (Figure 9 and Table 3), which is in agreement with previous findings in transgenic animals overexpressing *Ncx1*.^57^ The enhanced *Serca2a* expression and activity observed in AAV9.*miR‐1*–treated hearts together with the reduction in *Ncx1* expression results in a normalized *Ncx1*/*Serca2a* ratio, supporting a novel mechanism that *miR‐1* fine‐tunes the Ca^2+^ homeostasis in cardiomyocytes, suggesting that *miR‐1* plays a significant functional role in calcium metabolism in the heart.

**Table 3.** Ca^2+^ Transient Analysis

	AAB+Ad.GFP (n=48)	AAB+Ad.*miR‐1* (n=34)	*P* (*t* test)
Diastolic ([Ca^2+^]_i_)	0.923±0.026	0.923±0.043	0.94
Δ([Ca^2+^]_i_) (%)	17.610±4.81	17.454±3.73	0.87
Time to peak, ms	0.0683±0.005	0.0712±0.004	0.01
t_decay10_, ms	0.0368±0.005	0.0442±0.007	0.0001
t_decay50_, ms	0.134±0.024	0.1554±0.023	0.0001

Cardiomyocytes were isolated for pressure‐overload–induced hypertrophic hearts at 3 weeks post ascending aortic banding (AAB) and infected with an adenovirus expressing either *miR‐1* (Ad.*miR‐1*) or a control adenovirus (Ad.*GFP*). After 48 hours, the cardiomyocytes were loaded with a fluorescent Ca^2+^‐sensitive dye, Fura‐2, and the ratios of the fluorescence intensities (excited at 340 and 380 nm) were recorded using the IonOptix system. Diastolic ([Ca^2+^]_i_), Fura‐2 ratio at baseline; [Δ([Ca^2+^]_i_)], change in Fura‐2 ratio from baseline; t_decay10_, time for the transient 10% from peak; t_decay50_, time for the transient return 50% from peak.

It is possible that some of the beneficial effects observed in the *miR‐1*–treated hearts represents an indirect effect resulting from the reduced biomechanical overload secondary to the enhanced contractile function. In addition, we cannot exclude the possibility that *miR‐1* could directly affect signaling pathways in other cell types such as cardiac fibroblasts, smooth muscle cells, or endothelial cells. Nevertheless, regardless of the precise mechanism, the global changes induced by the restoration of *miR‐1* expression in the heart halted the progression of hypertrophy, ameliorating the pathological hypertrophic remodeling. Further investigation using the experimental platform used in this study has the potential to elucidate the specific mechanism(s) of miRNAs function in a disease setting. Future studies will provide a better understanding of the biological role of *miR‐1* in the development of heart failure and will lead to the identification of novel molecular effectors and signaling pathways that target the progression of maladaptive cardiac hypertrophy.

In conclusion, AAV‐mediated normalization of *miR‐1* expression ameliorates the hypertrophic phenotype and attenuates progressive deterioration of LV function through reversal of the cellular and molecular maladaptive remodeling associated with cardiac hypertrophy. Restoration of *miR‐1* levels, therefore, may lead to a novel therapeutic strategy to reverse cardiac hypertrophy.
